# The Medical Use of Electricity

**Published:** 1867-04

**Authors:** G. M. Beard, A. D. Rockwell

**Affiliations:** New York; New York


### THE MEDICAL USE OF ELECTRICITY.

By G. M. BEARD, M.D., and A. D. ROCKWELL, M.D., New York.

It was once remarked by a distinguished philosopher that we may be sure that a science is in its infancy when its advocates are particularly dogmatic in the expression of their beliefs.

Judged by this standard, the science of the medical use of electricity is just emerging from infancy into childhood, for its disciples are far less positive in the declaration of their views in regard to the unsettled points connected with it, than were the original investigators fifteen years ago. A brief survey of the history of recent explorations in this department will fully explain our meaning. In 1850, Duchenne, of Boulogne, published a work entitled, "Exposition d'une Nouvelle Methode de Galvanization, dite Galvanization Localiseé," in which he announced the leading idea of his system, namely, that "one can localize the electric stream over a fixed point under the skin, if the end of a conductor be covered with a moist spônge, and strongly pressed upon the skin." In 1855, he published another work, an epitome of five years experience, entitled, "De l'Electrization Localiseé et de son Application à la Physiologie, à la Pathologie, et à la Therapeutique." This book excited not a little attention, and really created an era in medical science. It aroused the enthusiasm of the author's countrymen, who had the opportunity of witnessing his brilliant demonstrations, and was abridged for the German physicians, by Dr. Erdmann. But the number of those who were inspired to enter the list as investigators was comparatively small. Like all the original explorers in this department, Duchenne laid special stress on his physiological experiments. At no era of medical science had there been found any considerable number in any county who were willing and able to devote their lives to purely experimental researches that achieve no direct practical results. While Duchenne had astonished both himself and the scientific world by the novelty of his experience, neither he nor his admirers were fully alive to the immense therapeutical value of his discoveries. As a consequence, the cause of electro-therapeutics would not be espoused by those who felt compelled to turn all their endeavors to practical account, and but very few of those who warmed into enthusiasm over his demonstrations were willing to occupy themselves exclusively in the same field. But Meyer of Berlin, Baierlacher of Nürnberg, Althaus of London, made a specialty of this department, and have published works in which the method of Duchenne is adopted as the basis. Remak, of Belin, was one of the few who were sufficiently alive to the value of Duchenne's experiments to pursue the subject still further, and in 1855, he embodied his experience in a work entitled, "*Ueber Methodische Electrisirung Gelahmter Muskeln*,"—"On the Methodical Electrization of Paralysed Muscles"—in which he announced a system entirely different from that of Duchenne, and became the leader of an opposite school. The leading idea of his work was this: "That in order to bring a muscle to complete contraction, it is far better to excite its motor nerves than to allow the stream to operate upon the muscular substance itself."

Moreover, he employed the galvanic stream almost entirely, while Duchenne had performed his experiments with the faradaic current. Remak reported astounding cures, but at first failed in enlisting the sympathy of the profession. Subsequently, however, he learned wisdom by experience, and became more wary in his statements, and calmer in his manner of expression. Gradually the profession accorded to him their confidence, and some of the ablest minds of Germany—among whom we may name Benedict, Schulz, Meyer, Neumann, Rosenthal, Frommhold, and Eulenberg—are numbered among his disciples. For some time a fierce and unreasonable controversy was carried on between Remak and Duchenne, which, both in its character and in its results, singularly reminds one of the fabled story of the knights who quarrelled over the pictures on the opposite side of the public sign. Remak contended for the application of the galvanic stream on the motor nerves, while Duchenne persisted that the best results were obtained with the faradaic, or induced current, on the muscles themselves.

At a public trial in Paris (as we are informed by Garratt), Remak demonstrated on a living subject that so far as producing contraction of a muscle goes, the induced current applied to the motor nerves is far less painful than when applied to the muscular substance. But so far as the general remedial effects of electricity are concerned, both were right, as is now tolerably well established.

So far as is yet ascertained, the remedial effects of electricity, whether employed in the form of galvanization or faradization, are governed by no fixed laws. Experience is our only guide in their use. All the investigations of scientific men have thus far failed in reducing to any system the different therapeutical effects of the galvanic and the faradic or induced currents.

Although there has been not a little speculation on this very theme, and although numberless experiments have been instituted with a view to establishing the superiority of the one or the other, yet we believe that the most progressive and candid of the investigators of the present day are willing to admit that the whole subject is yet in chaos. It has been found that the brilliant results that Remak obtained by the galvanic stream can also be secured by the use of the secondary current, and it has also been found that they are, so far as we can judge, very capricious in their effects, setting at defiance all the attempts of inquiring minds to reduce their employment to a logical system.

Those who are accustomed to use the various kinds of electricity, namely, the galvanic or constant stream from a large number of elements, and the primary and secondary induced current of the electro-magnetic machine, testify that while all are beneficial, *no one* has been found to be so superior to the others as to justify its exclusive use. It has been thought that the galvanic stream was more serviceable in rheumatic and neuralgic affections, and in paralysis dependent on central nerve derangements; but this is very far from being established

There are cases where at first the induced current acts more speedily than the galvanic stream, which cases, later on, seem to be more amenable to the latter. One day a patient may present himself with a rheumatic disorder that is at once relieved by faradization, and the next day another patient with precisely the same disease, at precisely the same stage, is more speedily aided by galvanization.

More than that, there are cases which, at the beginning of the *seance*, are benefited by galvanization, and before its close yield to faradization. Nay, further, there are instances where one form of electricity seems to irritate for the time being, while the other at once calms and soothes. What, then, is the law of their operation? There is none, so far as can be determined in the present state of our pathological knowledge, and there is no guide to their use except experience. The German investigators have carefully studied the effects of the different forms of electricity in exciting muscular contractions, and they have refined this subject, as it appears to us, to an absurb and profitless excess. Ziemssen has experimented largely on the dead subject, and has ascertained the particular border points that are most susceptible to the electrical influence.

It has been very clearly established that muscular contraction is produced more completely and with less pain when the electrodes are placed on the "border points" where the motor nerve enters the muscle, than when applied to the bellies of the muscles themselves.

With these views of Remak and Ziemssen our experience fully accords. We have found that in certain cases of atrophy and loss of power, where we fail in producing contractions by application directly over the muscles, we do succeed very unaccountably by pressing the fingers along and underneath the borders of the same muscles.

We have found that some inveterate cases of rheumatism that evinced no signs of yielding when the hand was applied in the general way, at once took a favorable turn when the fingers were pressed along and underneath the borders of the muscles concerned.

But important as is this fact, it is hardly worthy of the attention it has received at the hands of some of the German investigators.

Ziemssen and Rosenthal seem to act on the supposition that electricity is chiefly useful in cases of paralysis, whereas its proudest victories has been gained over long-standing dyspepsia, constipation, neuralgia, amenorrhœa, chorea, hysteria, and other disorders connected with enfeebled vital organs, and a deranged nervous system.

On the other hand, we have found that the different forms of paralysis that present themselves to us for treatment give far less speedy and satisfactory results to the electrical treatment than almost any other form of disease. And yet physicians and the layity are possessed with the idea that paralysis is, *par excellence*, the disease that justifies the use of the battery.—Our present knowledge of therapeutical value of the different forms of electricity may be thus summed up:

1st. Galvanization and faradaization are of so nearly equal value in paralytic, rheumatic, and neuralgic affections, that we are hardly justified in employing one to the exclusion of the other. It would seem that the best results are obtained when they are used with alternations and varations, according to the indication of their effects.

2d. Muscular contractions are excited with the least pain and most speedily when the electrode (either a sponge, a metal, or the hand) is applied along the edge of the muscle, and especially at the border point where the motor nerve enters the muscular substance.

3d. The ascending faradaic current has a great power of exciting muscular contractions, and stimulating the nerves, and is especially indicated in local paralysis, anchylosis, and plastic effusions. Applied through the body with the positive pole at the feet, its effects are not unfrequently very disagreeable.

4th. The *descending faradaic current, thoroughly applied, with the negative pole at the feet, is a tonic and corrective of far greater efficacy than any other remedy or combination of remedies now known to science.* Judiciously given, it can never work harm, save in cases of pulmonary tuberculosis, and in such cases we rarely employ it.

5th. The hand is by far the best electrode, and for the following reasons:

(*a*) It is more effective, from its wondrous power of adaptation. The fingers, better than sponge or metallic electrode, can feel their way along the border of the muscles and press towards the deeper tissues.

(*b*) By no other instrument can the patient receive the same quantity of electricity with so little irritatation, particularly when applied on the head, or over any sensitive diseased organ.

6th. *Although paralysis in its different forms is usually more benefited by electricity than by any system of internal medication, it is yet among the least tractable of the various diseases that present themselves for this method of treatment.*

7th. *The diseases which are found to yield most readily and surely to the different forms of electricity in the various modes of application, are neuralgia, dyspepsia, rheumatism of the subacute and chronic varieties, chronic bronchitis, constipation, amenorrhœa, anœmia, hysteria, and general debility.*

8th. The electic streams are of great value as adjuvants in the diagnosis of disease, inasmuch as any deviation from its normal sensitiveness of any part of their influence is readily indicated. In this way we learn where the disease is located, although we may not be able to determine its precise nature.—In order to illustrate more clearly to the profession the method and result of our treatment by faradaization, we propose to give in detail some of the more interesting cases of the various forms of disease in which we have employed it. We can hardly expect that the bare statement of the curative powers of electricity will receive absolute credence without the presentation of some typhical examples.

Even Remak himself (as stated above) at first experienced not a little difficulty in gaining the confidence of scientific men. So easy is it to represent on paper what is very difficult to practically achieve, and so ready are specialists to dignify their own peculiar method of treatment to the exclusion of all others, that we cannot hope for the full appreciation of the wondrous powers of electricity, except by those in the profession who may be led to employ the descending faradaic current as faithfully and persistently as we have ourselves.

*Neuralgia.*—This horrible affection, which so often disheartens both physician and patient, we have found to yield to the persistent application of electricity, as uniformly, perhaps, as any other disease, unless it may be nervous dyspepsia. Whether it exists either in the weekly or the strong it is always greatly alleviated and generally cured, sometimes by a few applications, in other cases by a protracted treatment.

Indeed, our unexpected success in certain cases did much to stimulate us to make special investigation on the whole subject of electricity.

Case First.—In the latter part of August of last year, Mr.

B——, a broker, down town, came to our office complaining of a severe neuralgia of the back of the head and neck, that had rendered his life miserable for one year and a-half. He had consulted many physicians and had taken much medicine, but had found little relief. He was a tall, well formed, muscular man, with a hard, full face, that suggested great native vigor of constitution.

With not very strong assurance of hope, either on our part or on his, the treatment by the descending faradaic current was recommended and commenced on the 5th of September. He was relieved on the first application, which was a very thorough one, and extended over all the vital organs. On the 8th of September he again came for treatment, and reported himself as much improved. On the 10th, he reported himself as being entirely free from pain. Another application was then made, and he bade us good-bye, preparatory to going into the country. We could hardly believe that the cure could be anything more than temporary, and were not surprised when he again presented himself on his return and informed us that his old enemy had made him another gentle visit.

We gave him another thorough application. A short time since he called at our office, and informed us that up to that time he had been perfectly well.

Case Second.—Miss H. The following case proves that that most distressing and persisting of all neuralgic troubles, tic douloureux, may be cured where medication had failed.

This patient presented herself for treatment in the early part of September, expressing herself, however, as having but little hope of experiencing more than a slight temporary relief.

Previously she had been treated by electricity with some short-lived benefit; sufficient, however, at that time to inspire her with hope. She was doomed to disappointment, for in a very few weeks the paroxysms of intense pain, which before had made her life miserable, returned with greater severity than ever. As she told her story, the sudden, sharp shooting pains in her face would, every few minutes, cause her in agony to hold her speech. To such an extent had this mysterious disease affected her that occasionally her tongue seemed almost paralyzed, and at times her utterance was thick and broken. These frequent and terrible paroxysms had left imprinted on her pale face an expression of constant care and suffering. Her own estimate of the value of life was that death was preferable to an existence of such constant agony. Her physician had tried almost everything in the materia medica supposed to be beneficial in such cases; and so without delay, doubting yet hoping, we commenced treatment by faradaization. The patient came every other day, and for the first two or three times only a comparatively mild current was given.

The strength was gradually increased, but no improvement was manifest. After the seventh or eighth application, however, a current, as strong as the operator and patient could well bear, was given for fifteen or twenty minutes, at each sitting, and in addition to the external application, the tongue was thoroughly electrified by means of a metallic spatula. From this time a rapid improvement was noticed.

The paroxysms occurred less frequently and with less severity. She discontinued treatment on the 20th of October, having had no return of pain for three weeks. Up to this date, January 2, she has had, so far as we can learn, no return of her trouble. Whether in this particular case the cure is *absolutely* permanent it is of course impossible to say; but enough is known to convince the patient and those who knew her that the benefit derived was great and incalculable.

Tic Douloureux, as it is well known by any who may have had much experience in the application of electricity for the disease, is the most difficult to cure or benefit of all neuralgic affections.

Of the neuralgias, the cervico-occipital, intercostal, lumbo-abdominial, and cephalalgia, are all in the vast majority of cases speedily cured; and although some cases of facial neuralgias, which have resisted all treatment by medication, still fail to be benefited by electricity, yet it is our experience that the great majority, even of such cases, are permanently cured by persistent treatment.

Case Third.—The power of electricity over neuralgia of a specific character, was very well illustrated in the case of a Mrs. B., sent to us by Dr. Cummings.

When she first consulted us she represented that for years she had been suffering from a terrible neuralgia in the head, and which at times attacked other parts of the body. For six weeks the pain had been so severe that she had been unable to pursue her usual avocation, which was that of an operator on a sewing machine. She was a plump, fleshy individual, with full round cheeks, indicative of anything but delicate health. Indeed, so vigorous did she seem, that we told her that only local applications would be necessary in her case, and accordingly directed her to place her hands on the sheet of copper to which the negative pole was attached, while a very gentle current was passed over the front and back part of the head. The application had not been continued more than two or three minutes, when she exclaimed, "Doctor! my pain is all gone; this is the first quiet moment I have had for a long time."

The sitting lasted about ten minutes, and at its close she was directed to come again on the following day. The next morning she informed us that she had experienced no more pain in her head, but that "the disease had gone to her breast and shoulders." This fact, taken in connection with some other statements that she had incidently made the day before, led us to suspect that syphilis was the cause of her sufferings. In reply to questions she confessed to every symptom of secondary syphilis, and, in fact, had been aware all along that the disease was in her system. According to our habit in such cases, we made the applications down the spine and over the vital organs. The relief was complete, although not as instantaneous as the day previous. Several times the applications were renewed at intervals of two or three days, and each time she expressed herself as very enthusiastic over the improvement she was making. We examined her throat with the laryngoscope, and found a slight degree of inflammation, which readily yielded to the nitrate of silver. The fifth time she came she reported that she had returned to her work, and was able to labor nearly as hard as at any time in her life. The horrible nocturnal pains had entirely disappeared, and her sleep, which before had been so much interrupted, was quiet and refreshing. There remained, however, some neuralgic distresses in the eyes, which disappeared after a few more local applications.—*Medical Record, N.Y.*

